# On the road to eliminate malaria in Sri Lanka: lessons from history, challenges, gaps in knowledge and research needs

**DOI:** 10.1186/1475-2875-13-59

**Published:** 2014-02-18

**Authors:** Nadira D Karunaweera, Gawrie NL Galappaththy, Dyann F Wirth

**Affiliations:** 1 Department of Parasitology, Faculty of Medicine, University of Colombo, Colombo, Sri Lanka; 2 Antimalaria Campaign, Ministry of Health, Colombo, Sri Lanka; 3 Department of Immunology and Infectious Diseases, School of Public Health, Harvard University, Boston, USA

**Keywords:** *Plasmodium*, *P. vivax*, *P. falciparum*, *Anopheles*, Eradication, Control, Tropical disease, Vector-borne, Mosquito, Asia

## Abstract

Malaria is one of the most important tropical diseases that has caused devastation throughout the history of mankind. Malaria eradication programmes in the past have had many positive effects but failed to wipe out malaria from most tropical countries, including Sri Lanka. Encouraged by the impressive levels of reduction in malaria case numbers during the past decade, Sri Lanka has launched a programme to eliminate malaria by year 2014. This article reviews the historical milestones associated with the malaria eradication programme that failed subsequently and the events that led to the launch of the ongoing malaria elimination plans at national-level and its strategies that are operational across the entire country. The existing gaps in knowledge are also discussed together with the priority areas for research to fill in these gaps that are posing as challenges to the envisaged goal of wiping out malaria from this island nation.

## Background

Over three billion people live at the risk of acquiring malaria worldwide [1], which mostly affects poor and vulnerable groups in tropical and subtropical areas, where the temperature and rainfall are conducive for development and spread of the causative parasite. History reveals that malaria is a scourge that led to relocation of people and even kingdoms in the past [2]. It spread wildly and literally uncontested before the advent of effective treatment to contain the parasite with the use of drugs, such as quinine, and chemicals (insecticides) to control the vector mosquito; dichloro-diphenyl-trichloroethane (DDT) being the first one.

### History of global malaria control efforts

Eradication of malaria was recognized as the ultimate goal of national control programmes by the eighth World Health Assembly (WHA) in 1955 [3]. A mathematical model that demonstrated the effective interruption of parasite transmission through adult mosquito control [4,5] formed the scientific basis for malaria eradication, while an effective tool for such transmission interruption appeared with the discovery of DDT as a potent residual insecticide in 1939. The logistical approach to eradication was based upon the particular outcomes of a malaria control campaign in Greece [6], with evidence of almost complete interruption of malaria transmission in selected areas [7]. These observations underpin the first important principle of the then global malaria eradication plan, which was that the complete interruption of malaria transmission for a few years in a defined area would enable discontinuation of insecticide spraying without the risk of a comeback [6]. However, the evidence of reappearance of *Anopheles sacharovi* shortly after spraying in a different part of Greece led to the suspicion of the appearance of DDT-resistance in mosquitoes, which was later confirmed through laboratory means [8]. This finding alerted the malaria community at that time that the major weapon in the campaign against malaria (DDT) would only be effective for a limited amount of time and therefore, the importance of a timeframe for the global malaria eradication effort. Based on the experiences of Greece, a five-year period was considered as being adequate (and cost-effective) to interrupt transmission, with the risk of insecticide resistance in mosquitoes increasing with usage beyond that prescribed period [7]. Therefore, a four-phased regimen for the implementation of national malaria eradication programmes was designed by the World Health Organization (WHO) Expert Committee on Malaria, which outlined a detailed plan that was limited in duration and geographic scope [6]. The plan consisted of a ‘preparatory phase’, when a National Malaria Eradication Service (NMES), was established to coordinate and execute all phases of the programme. This was followed by the ‘attack phase’ that began with the first round of total coverage spraying with the goal of complete interruption of malaria transmission by the fourth year of the ‘attack phase’ with an expected infant parasite rate of 0, annual parasite incidence of less than 0.5 cases per 1,000 population and case detection screening between 5-10% of the population. The attack phase was to be discontinued with the cessation of spraying when the surveillance data confirmed the interruption of transmission in a given region when it moved to the next phase, which was the ‘consolidation phase’. During the consolidation phase, intense surveillance was the primary task of the NMES, with the objective of rapid mopping up of any malaria cases that appeared before transmission could occur or to stop transmission as soon as possible. If no indigenous cases appeared for three years, malaria was recognized as been eradicated from that region. An official investigation by the WHO followed in order to confirm that the country had met the criteria outlined by the Eighth Report of the Expert Committee [9], which enabled certification of eradication to be awarded to that country and its inclusion in an official register of formerly malarious areas. At that point, the region would enter the maintenance phase, during which the NMES would be reintegrated into the National Health Service, which would assume responsibility for preventing the reemergence of malaria through rapid responses to any possible cases that appeared. This state of ‘constant vigilance’ was prescribed until the global eradication of malaria was achieved.

### Malaria and early control efforts in Sri Lanka

**Figure 1:**
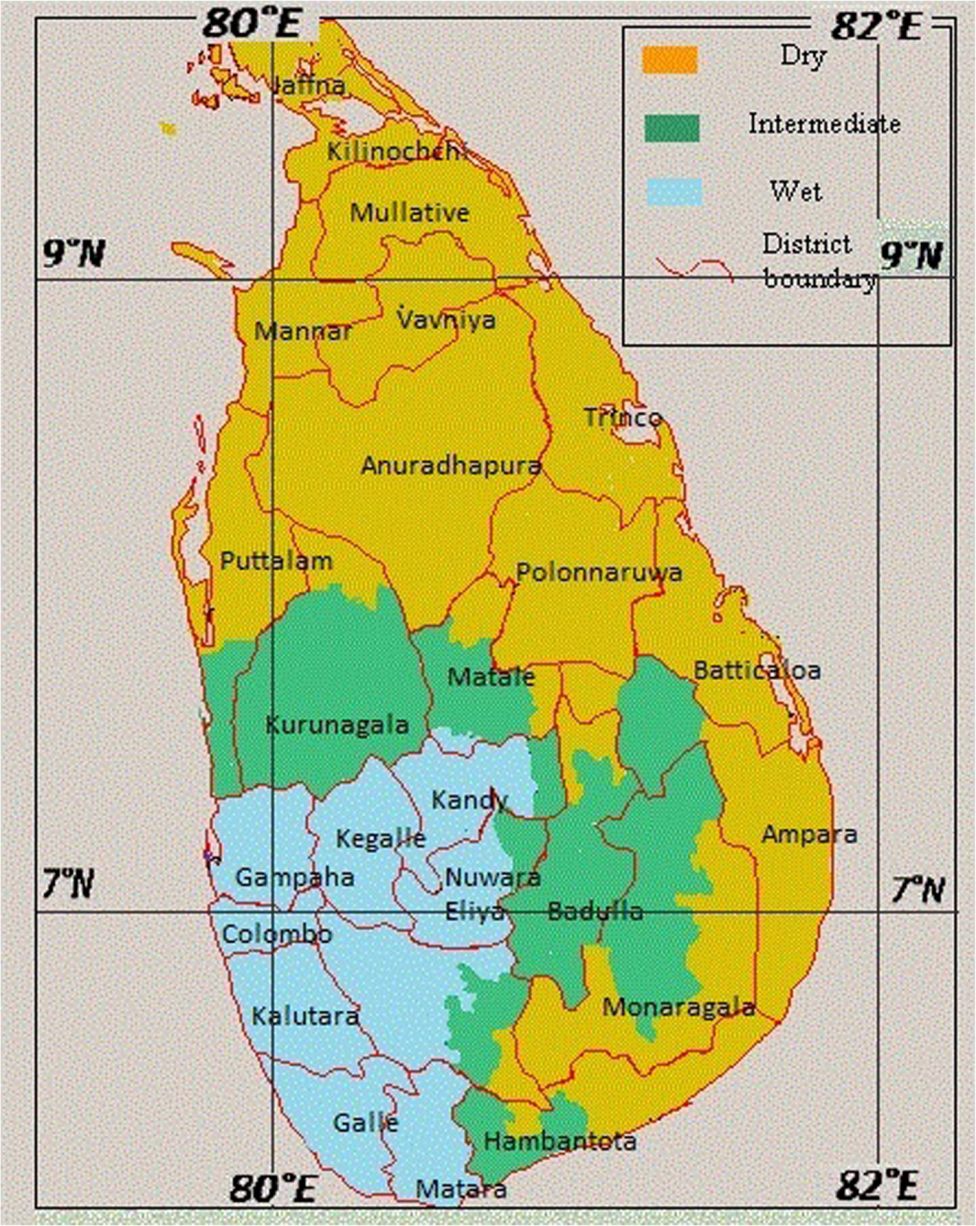
**Map of Sri Lanka.** Climatic zones (dry, intermediate and wet zones) of Sri Lanka, arbitrarily demarcated based on the annual rainfall.

Sri Lanka, known as Ceylon until 1972, is an island nation located off the southern coast of the Indian subcontinent in South Asia, positioned between latitudes 5° and 10°N and longitudes 79° and 82°E (Figure 1). The island is traditionally divided into three climatic zones viz. ‘dry’ , ‘intermediate’ and the ‘wet zone’ (Figure 1), based on seasonal rainfall. The wet zone receives relatively high mean annual rainfall of over 2,500 mm, particularly from the south-west monsoons (from April to June) and does not have any pronounced dry periods. The dry zone receives a mean annual rainfall of less than 1,750 mm, mostly through the north-east monsoons, which extends from October to January and a distinct dry season from May to September. The intermediate zone receives a mean annual rainfall between 1,750 to 2,500 mm with a short and less prominent dry season. The type of vegetation differs between these zones with south-western lowlands marked by the presence of dense rain forests, while tropical dry forests prevail in the dry zone.

Malaria endemicity in Sri Lanka varies along the climatic zones, and is determined by the habit of the principal vector mosquito, *Anopheles culicifacies*[10]. This species breeds in clean stagnant or slow moving waters. It typically thrives in the dry zone, where pools of water collect during the rainy season, and regularly invades the intermediate zones during periods of drought when rock and sand pools form along the banks of rivers and streams. Therefore, the traditional malaria-endemic zone extends through three quarters of the country encompassing most of the dry zone, with epidemic situations reported every now and then from the intermediate zone [11] (Figure 1). The vector and, as a result malaria, is rare in high altitude regions of the hill country (central part of the country) and in most of the wet zone except during periods of severe drought [12].

Vectors of malaria in Sri Lanka have been studied fairly extensively [10,13-20]. Behaviour of the principal vector *An. culicifacies* has already been well-documented, even before the beginning of control activities, by a number of entomological studies conducted throughout the 19^th^ and early 20^th^ centuries [21]. It is primarily a jungle species that is usually active from dusk until 9–10 pm and again towards dawn. The vector typically rests in dwellings after a blood meal [22-24], a habit that enables the successful use of residual insecticides.

**Figure 2:**
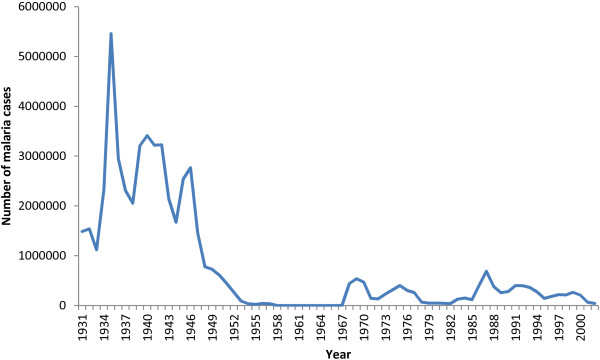
**Case incidence of malaria.** Case numbers recorded annually from 1931 to 2002 based on stained blood film examination for malaria parasites. Source of data: Anti-malaria campaign, Ministry of Health.

Sri Lanka, as a nation, had several features that facilitated the implementation of the then malaria eradication efforts. It is one of few tropical countries where a single principal vector (i.e. *An. culicifacies*) exists, whose behavioural characteristics render it poorly efficient as a vector and more susceptible to vector control measures [10]. Since it is an island nation with a contiguous landmass reintroduction of vectors from other nations is impeded. The efficient and well-organized postal system of the country enabled the daily dispatch and receipt of letters and parcels within 24 hours at the central laboratory from any part of the country therefore, allowed easy shipment of blood films and records for surveillance [23,25]. The massive epidemic of 1934/1935 [26] (Figure 2) may well have served as a major impetus in motivating both the government and the public to intensify control efforts and adoption of DDT spraying in 1945 [27], which led to a marked decline in the malaria case numbers (Figure 2) that in turn may have provided the necessary background and the confidence to initiate the malaria eradication program that was launched in 1958.

### Malaria eradication efforts in Sri Lanka (1958–1963)

With a declining trend in malaria transmission in Sri Lanka in the 1950s (Figure 2), which was in most part due to the use of indoor residual spraying of DDT, the adoption of an eradication programme was seen as a natural continuation of earlier efforts. Following the Eighth WHA resolution, the government accepted a proposal for a five-year programme of eradication in 1956 [24]. The programme was inaugurated in 1958 establishing the Anti-Malaria Campaign with its headquarters in Colombo [28]. The preparatory phase of the WHO plan was omitted in Sri Lanka due to a reasonably well-controlled malaria situation and infrastructure facilities that already existed in the country, and the dry zone was placed directly in ‘attack phase’, with the resumption of full coverage spraying. The intermediate and wet zones were placed in the ‘consolidation phase’ since there was no evidence of active transmission of malaria in those areas, and therefore, spraying was not considered as a requirement [29]. Entomological surveillance was intensified and prompt reporting of malaria cases was made a legal requirement. As a result of these efforts, annual parasite incidence (API) declined steadily between 1958 and 1963, even as the annual blood examination rate (ABER) quickly reached and exceeded the WHO recommendation of 5-10% [30]. An island-wide infant parasite survey was conducted in May, 1960 that included coverage of 20% of the infant population and the results confirmed zero prevalence of malaria [29] that was an early indication of the interruption of transmission. In 1963, parasite incidence reached a remarkably low-point with only 17 cases of malaria, which included only six indigenous cases (rest were imported) [31]. Given the favorable indicators, in April, 1963, spraying was halted throughout the country except for barrier spraying around jungle areas. Within months however, the appearance of new cases of malaria prompted the resumption of spraying in the affected areas [30]. Number of malaria cases continued to increase in the following years together with the vector density with over half a million cases of malaria reported by 1969 [31]. That year, a further setback occurred when DDT-resistance was discovered for the first time in Sri Lanka [27]. As resistance spread, the country switched to malathion in 1977 [32]. With the global campaign for eradication now over, the new insecticide served as the primary tool in a new control campaign [32]. But despite initial successes, malaria incidence continued to rise in the 1980’s (Figure 2). The control campaign faced yet a new challenge when chloroquine-resistant *Plasmodium falciparum* was discovered for the first time [33]. This was compounded by a prominent increase in *P. falciparum* cases and the discovery of new *P. falciparum* foci in the vicinity of newly constructed dams built to promote agriculture. New vector breeding pools were created on the river bed as a result of poor flow of water distal to the dam [34]. Though the exact reason for the failure of the eradication programme is unclear, multitude of reasons including parasite introduction through human migrations, asymptomatic parasite-carriers, vector-reintroduction, behavioural changes in the vector and the emergence of drug and insecticide resistance may have contributed. Unfortunately, very little documentation from this era remains and no parasite or vector material have been preserved for analysis.

### Recent history of malaria transmission and anti-malaria efforts

**Figure 3:**
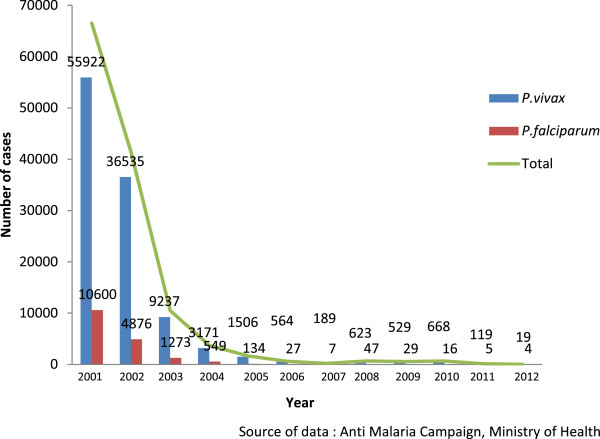
**Species distribution of malaria cases recorded in Sri Lanka from 2001 to 2012.** Incidence of recorded cases of *P. vivax*, *P. falciparum* and total number of cases in Sri Lanka from year 2001 up to 2012. From year 2010 onwards figures indicate the numbers of indigenous malaria cases. Source of data: Anti-malaria campaign, Ministry of Health.

**Figure 4:**
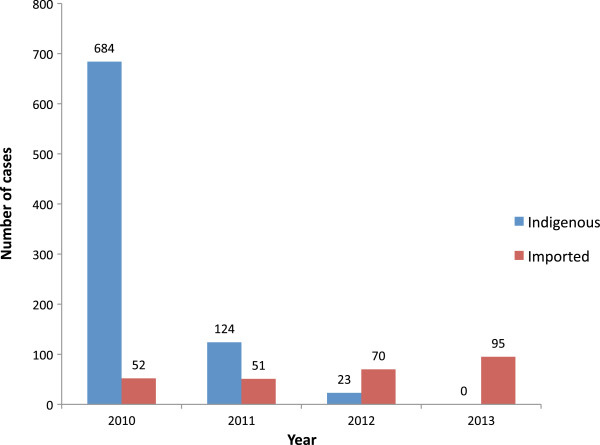
**Distribution of malaria cases reported (2010–2013).** Number of indigenous and imported cases of malaria recorded during years 2010, 2011, 2012 and 2013. Source of data: Anti-malaria campaign, Ministry of Health.

Incidence of malaria in Sri Lanka has markedly declined from year 2000 onwards, with a steady reduction in the proportion of *P. falciparum* cases (Figure 3). In fact, the recorded annual case numbers have been below 1000 since 2006 with the majority of cases due to *Plasmodium vivax* (Figure 3). During the years of 2011 and 2012 there were only 124 and 23 respectively of indigenous malaria cases. Remarkably, the numbers of *P. falciparum* cases during these years were limited to five (in 2011) and four (in 2012) (Figure 3). Furthermore, the majority (n = 99) reported in 2011 were personnel from the security forces who were engaged in post-civil war rehabilitation and reconstruction work in the northern and eastern parts of the country, which indicated the presence of pockets of active transmission at that time. Another notable feature during the last few years is the steady increase in the proportion/numbers of imported malaria cases (Figure 4) with India and Africa being the common source countries. This trend continues to date with no indigenous malaria cases reported during the year 2013.

### Plans for elimination of malaria in Sri Lanka

Global figures indicate that 109 countries are malaria-free [35] while 67 countries are still endemic for malaria with control efforts in place and 32 countries have adopted measures to eliminate malaria from within their borders [36]. Elimination is the total interruption of mosquito-borne malaria transmission in a geographically defined area. The malaria control programme of the Ministry of Health in Sri Lanka launched a ‘strategic plan for elimination of malaria’ in year 2008, building on the successful control efforts of preceding years [28]. Initial plans excluded the northern and eastern provinces due to the internal conflict situation that prevailed during that time. However, with the cessation of the civil war in 2009 the elimination drive was extended to cover the entire nation. At present, Sri Lanka is the only country in South Asia with such ambitious goals that in fact, the country has almost accomplished according to the national malaria surveillance data that continue to be rigorously collected at the headquarters of the anti-malaria campaign. Regular data flow to the headquarters occurs through their regional offices that spread across the country [28]. The objectives of the elimination drive include, elimination of indigenous *P. falciparum* malaria by year 2012, elimination of indigenous *P. vivax* malaria by 2014, maintenance of a zero mortality of malaria cases and prevention of re-introduction of malaria into the country [28].

Several strategies have been laid down (listed below) to achieve the stated objectives:

1. Early diagnosis and prompt treatment of malaria patients and asymptomatic parasite carriers

2. Implementation of selective and sustainable vector control measures based on the principles of integrated vector management protocols

3. Forecasting, early detection, prevention of outbreaks, and the rapid and effective containment of outbreaks

4. Regular reassessment of the country’s malaria situation and evaluation of malaria control activities

5. Enhancement of community participation and partnership building for effective and sustainable malaria control

6. Promotion of human resource development and capacity building

7. Promotion of operational research

### Challenges towards elimination

Majority of infections reported during the last few years (> 97%) are due to *P. vivax* (Figure 3). Primaquine that is given for 14 days is the only drug regimen available for radical cure and prevention of relapses in *P. vivax*. Achievement of 100% compliance for such a regimen remains as a challenge. Furthermore, sustenance of high-level of vigilance and clinical suspicion at the patients’ first point-of-contact, maintenance of good quality diagnostic skills and provision of prompt and effective treatment for malaria against the backdrop of low or negligible malaria case numbers are no easy tasks [37]. Maintenance of effective surveillance and response systems is recognized as an integral part of strategy to ensure success. However, the allocation of resources for these efforts are becoming more and more challenging with other public health issues with larger disease burdens e.g. dengue demanding more attention. Similarly, sustaining the necessary level of interest within the management/administration, field staff and more importantly the policy makers has become an enormous challenge with dwindling case numbers.

Country’s development projects following the war involve the presence of overseas workers, particularly from China and India. Presence of such a foreign labour force increases the risk of imported infections, including malaria. Similar risks have been identified in association with illegal migrants [38], security force personnel engaged in UN missions in malaria-endemic countries like Haiti and Sri Lankans who travel to other countries in search of jobs [39] or as tourists. Pilgrimage tours to India, which are popular among locals, pose yet another challenge increasing the risk of parasite carriage in to the country. It is obvious that the maintenance of the adopted strategies to achieve malaria elimination (and sustain thereafter) would require substantial funding and such monetary allocations in the midst of conflicting priorities for the government certainly is a challenge and will be more so in the future.

### Update on local parasite studies

The two prevalent parasite species in the island, viz. *P. vivax* and *P. falciparum* have been widely studied as to their genetic structure and diversity at varying time points and locations within the country. Genetic diversity of these *Plasmodium* species has been estimated by the investigation of allelic variation of polymorphic microsatellite loci [40-42] or vaccine candidate genes [43-48] or both types of markers [49]. Since the diversity of highly antigenic loci (vaccine candidates) reflects the combined effect of the parasite’s population history and selective constraints imposed by the host’s immunity [50] and therefore, is of limited value in providing information regarding the parasite population structure, neutral markers such as microsatellites, have provided more strong evidence for the presence of extensively diverse *P. vivax* parasite populations in Sri Lanka [51] with over 50% of mixed clonal infections [41], in spite of the relatively low and unstable transmission conditions that has prevailed during the past decade. Furthermore, in spite of the extensive haplotype diversity and multiclonal infections circulating in the population, the maintenance of linkage disequilibrium between physically unlinked loci, favours more of a clonal structure within the local parasite population, which is an interesting feature [41]. Studies of haplotype patterns that emerge following analyses of microsatellite data have demonstrated the usefulness of using these markers for mapping of focal outbreaks within the country [42] and to a limited extent for tracking the origin of the parasite isolates, a feature which would be of value during the elimination phase and even more so during the ‘prevention of re-introduction phase’ of malaria in any country [42]. Haplotype distribution and their likely origin with regard to known drug resistant markers also have been described in the local setting [52-56], some of which have influenced the changes in the national malaria drug policy, particularly against *P. falciparum* in the past.

### What is new regarding the vector in Sri Lanka?

Both past and present studies indicate that the principal vector of *P. vivax* and *P. falciparum* in Sri Lanka remains as *An. culicifacies*[10,57-59], which is considered as a zoophilic and indoor-resting mosquito. However, other anopheline species, such as *Anopheles subpictus, Anopheles annularis* have also been incriminated as vector species in the country [12,60,61]. Though stagnant or slow-moving natural pools with clear, sun-lit water collections were traditionally recognized as favourable breeding sites for the local *An. culicifacies* mosquitoes [10,62] more recent evidence points to its ability to adapt to variable conditions, such as increasing salinity in water [15] and even polluted water [63], which are important factors to consider from a vector control perspective. The role of different sibling species within the *Anopheles* species complexes in malaria transmission and their biological properties have also been reported through studies conducted in the endemic zones of the country [13,59,64]. Such knowledge regarding bio-ecological traits of sibling species, though still limited, has alerted the community of its potential implications and relevance of such considerations for the design and implementation of effective vector control measures, particularly during this critical phase of malaria elimination.

### Gaps in knowledge, research and operational needs

Malaria case numbers have remained low in Sri Lanka during the past decade or so, which fully justifies the country’s efforts towards eliminating this menace. The few cases reported are almost exclusively due to *P. vivax*, while *P. falciparum* remains rare and predominantly a disease of travelers. Detailed investigations, including genetic typing and characterization, and material archiving of each and every parasite isolate that appears during this period would be of great value to inform the present as well as to enable further investigations in the future. Therefore, the importance of maintaining a parasite bank with a well-characterized parasite database for all identified cases cannot be emphasized enough.

With the cessation of war in 2009 the government’s plans for the country’s development have focused more and more on promoting Sri Lanka as a top tourist destination. With more frequent travel of both the tourists and the locals, ‘imported malaria’ has become the most important category in determining the malaria case incidence in present day Sri Lanka. The primary source of the foci of malaria transmission that appear occasionally in the dry zone (that remains conducive for its transmission) and almost exclusively due to *P. vivax* is difficult to ascertain. Source of *P. vivax* that emerges in such a setting could be either a relapsed parasite or a new introduction. Therefore, in this backdrop the availability of genetic tools that could enable fine tracking of source parasites would be invaluable. Microsatellite markers in *P. vivax,* which are neutral and rapidly evolving [40,65], have been tested and used for the study of geographical clustering of patient isolates [42] with early evidence indicating their usefulness in tracking of the source of parasites, at least to regional level [42]. However, it appears that more sensitive tools e.g. single nucleotide polymorphisms (SNPs) need to be developed and tested that would assist in such fine-level tracking of parasite isolates in an elimination setting. Such studies have already been done for *P. falciparum*[66]. Therefore, equally sensitive tools for tracking of *P. vivax* remains as a priority research need for all elimination settings.

Presence of asymptomatic carriers of parasites, especially gametocyte-carriers remains a true possibility in traditionally endemic zones. However, previous attempts to detect such asymptomatic parasitaemias have not been successful [67,68]. The apparent absence of asymptomatic parasite carriage and the obvious conclusions drawn from such studies however, are limited by the level of sensitivity of the tools used for parasite detection. Therefore, better tools for detection of low-level parasitaemia, including gametocytes that could act as triggers for transmission foci within the island, remain as a research need.

Sero-prevalence rates of anti-malarial antibodies have been used as a reliable tool to assess malaria endemicity in different settings [69,70]. A study done in 2006/2007 in a previously malaria-endemic area in the south of Sri Lanka demonstrated the persistence of high levels of anti-malarial antibodies in majority of residents, in spite of the very low malaria case numbers reported during preceding years [71]. Therefore, the estimation of sero-prevalence rates could be a useful tool to validate and test in the local setting, which may be of value for the verification of the present status of elimination.

Furthermore, attention also should be drawn towards the need for maintenance of high-quality laboratory diagnostic services, both in the state and the private sector, to ensure prompt and accurate diagnosis of malaria. Though rapid diagnostic tests are indeed used in the health sector, due to the high cost and limited availability, microscopy remains as an important tool particularly in the state sector laboratories. With scanty case numbers seen in the country during the past several years there has been an obvious decline of skills and competencies of laboratory personnel. Therefore, mechanisms should be put in place to ensure internal and external quality assurance programmes for malaria diagnostic laboratories with opportunities for regular training of laboratory staff.

Vector control, particularly through the use of adulticides, remains as an important strategy in targeted settings with evidence of malaria transmission. Changing weather patterns, ecosystems within the island and post-war infrastructure development projects are just few factors that are likely to influence the vector breeding habits and their abundance. Furthermore, the potential risk of introduction of exotic vectors through importation is also an aspect that should not be overlooked. Therefore, studies on vectors and their behaviours remain important in order to gain knowledge on possible variations that could have implications on effective vector control in an elimination setting. Furthermore, important vector indices for malaria transmission such as species prevalence and vector density variations should continue to be monitored and diligently recorded.

Drug policies adopted since commencement of malaria elimination strategies in Sri Lanka for both *P. vivax* (chloroquine and primaquine) and *P. falciparum* (Coartem® and primaquine) have been maintained island-wide without any deviations or revisions. Careful post-treatment follow-up of patients remains critically important to enable early detection of poor therapeutic response, if any and provide opportunities for timely interventions. With the persistent risk of imported malaria, in addition to the routine screening offered at ports of entry, innovative strategies such as interactive networks between countries for prompt sharing of data on disease outbreaks and drug-susceptible patterns should be put in place, in order to identify the need for timely review of surveillance strategies/intensities and drug policies in the local setting. Since majority of reported infections do remain as *P. vivax,* development of an effective chemotherapeutic agent (more so than currently used drug for this purpose, primaquine) that abolishes hypnozoites and resultant relapses is an important need for the future. Studies towards such a goal remain a priority not only for Sri Lanka but for all countries with plans for malaria elimination. The practice of primaquine usage that is being continued to date to enable radical cure of *P. vivax* has brought out the concerns within the medical community of the risk of haemolysis in G6PD-deficient patients, which is a complication that has been reported from malaria endemic areas in the past [72]. Available data on G6PD-deficiency among Sri Lankans are several decades old [73,74] and grossly inadequate with estimated prevalence of functional enzyme deficiency ranging between 1% and 5%. Current tools could be made use of to update the knowledge in this area, which in turn would enable more accurate assessment of the burden of this condition and therefore, help in the design of strategies for prevention of potential risks associated with anti-malarial therapy in affected individuals.

## Conclusions

There are indeed absolute benefits in achieving elimination of malaria in Sri Lanka, not the least of which is ensuring the end of human suffering caused by this disease. The pathway of elimination, however, requires both patience and constant ingenuity in surveillance with close monitoring, timely adjustment of strategies, intelligent use of available tools to assist the process, development and testing of more tools to compete against a changing parasite/vectors and which will help to overcome the associated hurdles encountered during the challenging, but promising road, towards malaria elimination. All stakeholders should remain convinced that to achieve and thereafter, to maintain the status of elimination of malaria would continue to demand substantial amounts of resources that, however, will be offset by the multitude of resultant benefits that will be accrued by the country and its citizens.

Globalization in particular calls into question the idea of a defensible, geographically enclosed territory against infectious disease. It may not be realistic to expect prolonged sustenance of the intensity of efforts that are being practiced since the launch of the elimination programme, which has enabled the achievement of an almost malaria-free status within the borders of Sri Lanka. This highlights the need for more globally coordinated approaches to achieve and maintain a malaria-free status even in a given geographically-defined setting.

Finally, an examination of the history of malaria eradication in Sri Lanka argues for the value of intense efforts in using scientifically valid and up-to-date methods to monitor parasites, vectors and for the study of socio-cultural aspects and more importantly, the careful maintenance of a database with proper preservation of parasite/vector material that would enable further investigations if and when necessary. There is still a lack of understanding of the precise biology behind the resurgence of malaria following near-eradication in the 1960s, which include the relative roles of undetected foci of infection, introduction of parasites through human movements, vector reintroduction, changes in vector behaviour, human migration, imported human hosts, and the emergence of drug resistance, all of which are worth exploring in greater detail during the ongoing elimination phase of malaria, particularly since there are better tools available to do so now. This underscores the need for integrating new technologies in the process of assessing the present and informing the future in order to achieve successful elimination of malaria in Sri Lanka.

## Abbreviations

DDT: Dichloro-diphenyl-trichloroethane; NMES: National malaria eradication service; WHA: World health assembly; WHO: World Health Organization.

## Competing interests

The authors declare that they have no competing interests.

## Authors’ contributions

NDK drafted the manuscript, acquired data and carried out the analysis. GNLG drafted a section of the article and contributed data. DFW provided comments for improvement of the manuscript. All authors read and approved the final manuscript.

## References

[B1] WHO World Malaria Report 2013 2013 Geneva: World Health Organization

[B2] Carter R Mendis KN Evolutionary and historical aspects of the burden of malaria Clin Microbiol Rev 2002 15 564 594 10.1128/CMR.15.4.564-594.2002 12364370 PMC126857

[B3] WHO Malaria. Handbook of resolutions and decisions of the World Health Assembly and the Executive Board 1973 Geneva: World Health Organization

[B4] Macdonald G The epidemiology and control of malaria 1957 London: Oxford University Press

[B5] Macdonald G Epidemiological basis of malaria control Bull World Health Organ 1956 15 613 626 13404439 PMC2538278

[B6] Pampana E A textbook of malaria eradication 1963 Oxford: Oxford University Press

[B7] Livadas GA Belios G Postwar malaria control in Greece and its results on basis of epidemiological data. WHO/Mal/27 1948 Geneva: World Health Organization WHO/Mal/27 18872937

[B8] Brown AWA The insecticide resistance problem Bull World Health Organ 1958 18 309 321 13536795 PMC2537653

[B9] WHO Expert Committee on Malaria Eighth Report World Health Organization technical report series 1961 Geneva: World Health Organization 14447795

[B10] Amerasinghe PH Amerasinghe FP Konradsen F Fonseka KT Wirtz RA Malaria vectors in a traditional dry zone village in Sri Lanka Am J Trop Med Hyg 1999 60 421 429 10466971 10.4269/ajtmh.1999.60.421

[B11] Ramasamy R Ramasamy MS Wijesundera DA Wijesundera AP Dewit I Ranasinghe C Srikrishnaraj KA Wickremaratne C High seasonal malaria transmission rates in the intermediate rainfall zone of Sri Lanka Ann Trop Med Parasitol 1992 86 591 600 1304700 10.1080/00034983.1992.11812714

[B12] Amerasinghe PH Amerasinghe FP Wirtz RA Indrajith NG Somapala W Pereira LR Rathnayake AM Malaria transmission by *Anopheles subpictus* (Diptera: Culicidae) in a new irrigation project in Sri Lanka J Med Entomol 1992 29 577 581 1495065 10.1093/jmedent/29.4.577

[B13] De Silva BG Gunasekera MB Abeyewickreme W Abhayawardana TA Karunanayake EH Screening of *Anopheles culicifacies* population of Sri Lanka for sibling species A Indian J Malariol 1998 35 1 7 10319555

[B14] Amerasinghe PH Amerasinghe FP Multiple host feeding in field populations of *Anopheles culicifacies* and *An. subpictus* in Sri Lanka Med Vet Entomol 1999 13 124 131 10.1046/j.1365-2915.1999.00160.x 10484158

[B15] Jude PJ Dharshini S Vinobaba M Surendran SN Ramasamy R *Anopheles culicifacies* breeding in brackish waters in Sri Lanka and implications for malaria control Malar J 2010 9 106 10.1186/1475-2875-9-106 20409313 PMC2864285

[B16] Surendran SN Singh OP Jude PJ Ramasamy R Genetic evidence for malaria vectors of the *Anopheles sundaicus* complex in Sri Lanka with morphological characteristics attributed to *Anopheles subpictus* species B Malar J 2010 9 343 21114832 10.1186/1475-2875-9-343 PMC3009661

[B17] Kannathasan S Antonyrajan A Srikrishnaraj KA Karunaratne SH Karunaweera ND Surendran SN Studies on prevalence of anopheline species and community perception of malaria in Jaffna district, Sri Lanka J Vector Borne Dis 2008 45 231 239 18807380

[B18] Perera MD Hemingway J Karunaratne SP Multiple insecticide resistance mechanisms involving metabolic changes and insensitive target sites selected in anopheline vectors of malaria in Sri Lanka Malar J 2008 7 168 10.1186/1475-2875-7-168 18755020 PMC2547111

[B19] Kusumawathie PH Wickremasinghe AR Karunaweera ND Wijeyaratne MJ Yapabandara AM Anopheline breeding in river bed pools below major dams in Sri Lanka Acta Trop 2006 99 30 33 10.1016/j.actatropica.2006.06.007 16890181

[B20] Premasiri DA Wickremasinghe AR Premasiri DS Karunaweera N Malarial vectors in an irrigated rice cultivation area in southern Sri Lanka Trans R Soc Trop Med Hyg 2005 99 106 114 10.1016/j.trstmh.2004.02.009 15607337

[B21] Malaria Atlas Project [http://www.map.ox.ac.uk/explore/countries/LKA/#!mosquito-vectors]

[B22] Rawlings P Curtis CF Tests for the existence of genetic variability in the tendency of *Anopheles culicifacies* species B to rest in houses and to bite man Bull World Health Organ 1982 60 427 432 6982776 PMC2535996

[B23] Rajendram S Abdul Cader MH Visvalingam T Malaria eradication in Ceylon Nature 1950 166 486 14780109 10.1038/166486a0

[B24] Wickramasinghe MB Malaria and its control in Sri Lanka Ceylon Med J 1981 26 107 115 6764384

[B25] Visvalingam T The laboratory organization and technical procedures for blood examination of the malaria eradication programme in Ceylon WHO/Mal/419 1963 1 12

[B26] Jones M The Ceylon malaria epidemic of 1934–35: a case study in colonial medicine Soc Hist Med 2000 13 87 109 10.1093/shm/13.1.87 11624427

[B27] Pinikahana J Dixon RA Trends in malaria morbidity and mortality in Sri Lanka Indian J Malariol 1993 30 51 55 8405594

[B28] Anti malaria campaign Sri Lanka [http://www.malariacampaign.gov.lk/precentation/AboutUs.aspx]

[B29] WHO Development of the malaria eradication programme Fourteenth World Health Assembly report 1968 World Health Organization, Technical Report Series No. 382

[B30] Sivaghanasundram C Dynamics of malaria in Ceylon MD thesis 1971 London School of Hygiene and Tropical Medicine

[B31] Antimalaria Campaign Ministry of Health, Sri Lanka Administrative repot 1963 Sri Lanka: Antimalaria Campaign, Ministry of Health

[B32] Herath PR Joshi GP Pesticide selection pressure on *Anopheles subpictus* in Sri Lanka: comparison with two other Sri Lankan anophelines Trans R Soc Trop Med Hyg 1989 83 565 567 10.1016/0035-9203(89)90298-8 2617615

[B33] Ratnapala R Subramaniam K Yapabandara MG Fernando WP Chloroquine resistant *Plasmodium falciparum* in Sri Lanka Ceylon Med J 1984 29 135 145 6398762

[B34] Wijesundera Mde S Malaria outbreaks in new foci in Sri Lanka Parasitol Today 1988 4 147 150 10.1016/0169-4758(88)90193-7 15463072

[B35] Feachem RGA Shrinking the malaria map: a guide on malaria elimination for policy makers 2009 San Francisco: University of California

[B36] Feachem RG Phillips AA Targett GA Snow RW Call to action: priorities for malaria elimination Lancet 2010 376 1517 1521 10.1016/S0140-6736(10)61500-0 21035844 PMC3513636

[B37] Premaratna R Galappaththy G Chandrasena N Fernando R Nawasiwatte T de Silva NR de Silva HJ What clinicians who practice in countries reaching malaria elimination should be aware of: lessons learnt from recent experience in Sri Lanka Malar J 2011 10 302 10.1186/1475-2875-10-302 21999636 PMC3216287

[B38] Wickramage K Galappaththy GN Malaria burden in irregular migrants returning to Sri Lanka from human smuggling operations in West Africa and implications for a country reaching malaria elimination Trans R Soc Trop Med Hyg 2013 107 337 340 10.1093/trstmh/trt009 23584376

[B39] Sri Lanka Bureau of Foreign Employment [http://www.slbfe.lk/article.php?article=68]

[B40] Karunaweera ND Ferreira MU Hartl DL Wirth DF Fourteen polymorphic microsatellite DNA markers for the human malaria parasite *Plasmodium vivax* Mol Ecol Notes 2007 7 172 175

[B41] Karunaweera ND Ferreira MU Munasinghe A Barnwell JW Collins WE King CL Kawamoto F Hartl DL Wirth DF Extensive microsatellite diversity in the human malaria parasite *Plasmodium vivax* Gene 2008 410 105 112 10.1016/j.gene.2007.11.022 18226474

[B42] Gunawardena S Karunaweera ND Ferreira MU Phone-Kyaw M Pollack RJ Alifrangis M Rajakaruna RS Konradsen F Amerasinghe PH Schousboe ML Galappaththy GNL Abeyasinghe RR Hartl DL Wirth DF Geographic structure of Plasmodium vivax: microsatellite analysis of parasite populations from Sri Lanka, Myanmar, and Ethiopia Am J Trop Med Hyg 2010 82 235 242 10.4269/ajtmh.2010.09-0588 20133999 PMC2813164

[B43] Gunasekera AM Wickramarachchi T Neafsey DE Ganguli I Perera L Premaratne PH Hartl D Handunnetti SM Udagama-Randeniya PV Wirth DF Genetic diversity and selection at the *Plasmodium vivax* apical membrane antigen-1 (PvAMA-1) locus in a Sri Lankan population Mol Biol Evol 2007 24 939 947 10.1093/molbev/msm013 17244598

[B44] Dias S Somarathna M Manamperi A Escalante AA Gunasekera AM Udagama PV Evaluation of the genetic diversity of domain II of *Plasmodium vivax* Apical Membrane Antigen 1 (PvAMA-1) and the ensuing strain-specific immune responses in patients from Sri Lanka Vaccine 2011 29 7491 7504 10.1016/j.vaccine.2011.07.029 21784116

[B45] Premaratne PH Aravinda BR Escalante AA Udagama PV Genetic diversity of *Plasmodium viva*x Duffy Binding Protein II (PvDBPII) under unstable transmission and low intensity malaria in Sri Lanka Infect Genet Evol 2011 11 1327 1339 10.1016/j.meegid.2011.04.023 21554998

[B46] Dias S Longacre S Escalante AA Udagama-Randeniya PV Genetic diversity and recombination at the C-terminal fragment of the merozoite surface protein-1 of *Plasmodium vivax* (PvMSP-1) in Sri Lanka Infect Genet Evol 2011 11 145 156 10.1016/j.meegid.2010.09.007 20933611

[B47] Wickramarachchi T Premaratne PH Dias S Handunnetti SM Udagama-Randeniya PV Genetic complexity of *Plasmodium vivax* infections in Sri Lanka, as reflected at the merozoite-surface-protein-3alpha locus Ann Trop Med Parasitol 2010 104 95 108 10.1179/136485910X12607012374190 20406577

[B48] Manamperi A Mahawithanage S Fernando D Wickremasinghe R Bandara A Hapuarachchi C Wickremasinghe R Genotyping of *Plasmodium vivax* infections in Sri Lanka using Pvmsp-3α and Pvcs genes as markers: a preliminary report Trop Biomed 2008 25 100 106 18948880

[B49] Schousboe ML Rajakaruna RS Amerasinghe PH Konradsen F Ord R Pearce R Bygbjerg IC Roper C Alifrangis M Analysis of polymorphisms in the merozoite surface protein-3alpha gene and two microsatellite loci in Sri Lankan *Plasmodium vivax:* evidence of population substructure in Sri Lanka Am J Trop Med Hyg 2011 85 994 1001 10.4269/ajtmh.2011.11-0338 22144433 PMC3225177

[B50] Escalante AA Cornejo OE Rojas A Udhayakumar V Lal AA Assessing the effect of natural selection in malaria parasites Trends Parasitol 2004 20 388 395 10.1016/j.pt.2004.06.002 15246323

[B51] Gunawardena SG Ferreira MU Kapilananda GMG Wirth DF Karunaweera ND The Sri Lankan paradox: high genetic diversity in *Plasmodium vivax* populations despite decreasing levels of malaria transmission Parasitology 2014 doi:10.1017/S0031182013002278 10.1017/S0031182013002278 PMC7485621 24533989

[B52] Hapuarachchi HC Dayanath MY Bandara KB Abeysundara S Abeyewickreme W de Silva NR Hunt SY Sibley CH Point mutations in the dihydrofolate reductase and dihydropteroate synthase genes of *Plasmodium falciparum* and resistance to sulfadoxine-pyrimethamine in Sri Lanka Am J Trop Med Hyg 2006 74 198 204 16474070

[B53] Schousboe ML Rajakaruna RS Salanti A Hapuarachchi HC Galappaththy GN Bygbjerg IC Amerasinghe PH Konradsen F Alifrangis M Island-wide diversity in single nucleotide polymorphisms of the Plasmodium vivax dihydrofolate reductase and dihydropteroate synthetase genes in Sri Lanka Malar J 2007 6 28 10.1186/1475-2875-6-28 17349045 PMC1831779

[B54] Hawkins VN Auliff A Prajapati SK Rungsihirunrat K Hapuarachchi HC Maestre A O’Neil MT Cheng Q Joshi H Na-Bangchang K Sibley CH Multiple origins of resistance-conferring mutations in *Plasmodium vivax* dihydrofolate reductase Malar J 2008 7 72 10.1186/1475-2875-7-72 18442404 PMC2383903

[B55] Hapuarachchi HC Abeysundara S Dayanath MY Manamperi A Abeyewickreme W de Silva NR Molecular markers of chloroquine resistance in *Plasmodium falciparum* in Sri Lanka: frequency before revision of the antimalarial drug policy Ann Trop Med Parasitol 2009 103 351 356 10.1179/136485909X435067 19508753

[B56] Zhang JJ Senaratne TN Daniels R Valim C Alifrangis M Amerasinghe P Konradsen F Rajakaruna R Wirth DF Karunaweera ND Distribution pattern of *Plasmodium falciparum* chloroquine transporter (pfcrt) gene haplotypes in Sri Lanka 1996–2006 Am J Trop Med Hyg 2011 85 811 814 10.4269/ajtmh.2011.11-0167 22049031 PMC3205623

[B57] Rawlings P Herath PR Kelly S *Anopheles culicifacies* (Diptera: Culicidae): DDT resistance in Sri Lanka prior to and after cessation of DDT spraying J Med Entomol 1985 22 361 365 4045932 10.1093/jmedent/22.4.361

[B58] Surendran SN Ramasamy R Some characteristics of the larval breeding sites of *Anopheles culicifacies* species B and E in Sri Lanka J Vector Borne Dis 2005 42 39 44 16161699

[B59] Surendran SN Ramasamy MS De Silva BG Ramasamy R *Anopheles culicifacies* sibling species B and E in Sri Lanka differ in longevity and in their susceptibility to malaria parasite infection and common insecticides Med Vet Entomol 2006 20 153 156 10.1111/j.1365-2915.2006.00601.x 16608500

[B60] Surendran SN Jude PJ Ramasamy R Variations in salinity tolerance of malaria vectors of the *Anopheles subpictus* complex in Sri Lanka and the implications for malaria transmission Parasit Vectors 2011 4 117 10.1186/1756-3305-4-117 21702917 PMC3141743

[B61] Surendran SN Gajapathy K Kumaran V Tharmatha T Jude PJ Ramasamy R Molecular evidence for the presence of malaria vector species a of the *Anopheles annularis* complex in Sri Lanka Parasit Vectors 2011 4 239 10.1186/1756-3305-4-239 22192337 PMC3293028

[B62] Van Der Hoek W Konradsen F Amerasinghe PH Perera D Piyaratne MK Amerasinghe FP Towards a risk map of malaria for Sri Lanka: the importance of house location relative to vector breeding sites Int J Epidemiol 2003 32 280 285 10.1093/ije/dyg055 12714550

[B63] Gunathilaka N Fernando T Hapugoda M Wickremasinghe R Wijeyerathne P Abeyewickreme W *Anopheles culicifacies* breeding in polluted water bodies in Trincomalee District of Sri Lanka Malar J 2013 12 285 10.1186/1475-2875-12-285 23958454 PMC3765073

[B64] Surendran SN Jude PJ Weerarathne TC Parakrama Karunaratne SH Ramasamy R Variations in susceptibility to common insecticides and resistance mechanisms among morphologically identified sibling species of the malaria vector Anopheles subpictus in Sri Lanka Parasit Vectors 2012 5 34 10.1186/1756-3305-5-34 22325737 PMC3317438

[B65] Anderson TJ Haubold B Williams JT Estrada-Franco JG Richardson L Mollinedo R Bockarie M Mokili J Mharakurwa S French N Whitworth J Velez ID Brockman AH Nosten F Ferreira MU Day KP Microsatellite markers reveal a spectrum of population structures in the malaria parasite *Plasmodium falciparum* Mol Biol Evol 2000 17 1467 1482 10.1093/oxfordjournals.molbev.a026247 11018154

[B66] Daniels R Volkman SK Milner DA Mahesh N Neafsey DE Park DJ Rosen D Angelino E Sabeti PC Wirth DF Wiegand RC A general SNP-based molecular barcode for *Plasmodium falciparum* identification and tracking Malar J 2008 7 223 10.1186/1475-2875-7-223 18959790 PMC2584654

[B67] Fernando SD Abeyasinghe RR Galappaththy GN Rajapaksa LC Absence of asymptomatic malaria infections in previously high endemic areas of Sri Lanka Am J Trop Med Hyg 2009 81 763 767 10.4269/ajtmh.2009.09-0042 19861607

[B68] Rajakaruna RS Alifrangis M Amerasinghe PH Konradsen F Pre-elimination stage of malaria in Sri Lanka: assessing the level of hidden parasites in the population Malar J 2010 9 25 10.1186/1475-2875-9-25 20089157 PMC2818647

[B69] Drakeley CJ Corran PH Coleman PG Tongren JE McDonald SL Carneiro I Malima R Lusingu J Manjurano A Nkya WM Lemnge MM Cox J Reyburn H Riley EM Estimating medium- and long-term trends in malaria transmission by using serological markers of malaria exposure Proc Natl Acad Sci U S A 2005 102 5108 5113 10.1073/pnas.0408725102 15792998 PMC555970

[B70] Corran P Coleman P Riley E Drakeley C Serology: a robust indicator of malaria transmission intensity? Trends Parasitol 2007 23 575 582 10.1016/j.pt.2007.08.023 17988945

[B71] Dewasurendra RL Suriyaphol P Fernando SD Carter R Rockett K Corran P Kwiatkowski D Karunaweera ND Genetic polymorphisms associated with anti-malarial antibody levels in a low and unstable malaria transmission area in southern Sri Lanka Malar J 2012 11 281 10.1186/1475-2875-11-281 22905743 PMC3459805

[B72] Abeyaratne KP Halpe NL Sensitivity to primaquine in Ceylonese children due to deficiency of erythrocytic glucose-6-phosphate dehydrogenase Ceylon Med J 1968 13 134 138 5721557

[B73] Abeyaratne KP Premawansa S Rajapakse L Roberts DF Pipiha SS A survey of glucose-6-phosphate-dehydrogenase deficiency in the North Central Province of Sri Lanka (formerly Ceylon) Am J Phys Anthropol 1976 44 135 138 10.1002/ajpa.1330440119 1247108

[B74] Nagaratnam N Leelawathie PK Weerasinghe WM Enzyme glucose-6 phosphate dehydrogenase (G6PD) deficiency among Sinhalese in Ceylon as revealed by the methaemoglobin reduction test Indian J Med Res 1969 57 569 572 5824012

